# Serum proteomic profiling of patients with compensated advanced chronic liver disease with and without clinically significant portal hypertension

**DOI:** 10.1371/journal.pone.0301416

**Published:** 2024-04-11

**Authors:** Frane Pastrovic, Rudjer Novak, Ivica Grgurevic, Stela Hrkac, Grgur Salai, Marko Zarak, Lovorka Grgurevic

**Affiliations:** 1 Department of Gastroenterology, Hepatology and Clinical Nutrition, Laboratory for Liver Diseases and Portal Hypertension, University Hospital Dubrava, Zagreb, Croatia; 2 Faculty of Pharmacy and Biochemistry, University of Zagreb, Zagreb, Croatia; 3 Department of Proteomics, Center for Translational and Clinical Research, University of Zagreb School of Medicine, Zagreb, Croatia; 4 University of Zagreb, School of Medicine, Zagreb, Croatia; 5 Biomedical Research Center Salata, University of Zagreb, School of Medicine, Zagreb, Croatia; 6 Department of Clinical Immunology, Allergology and Rheumatology, University Hospital Dubrava, Zagreb, Croatia; 7 Department of Pulmonology, University Hospital Dubrava, Zagreb, Croatia; 8 Clinical Department of Laboratory Diagnostics, University Hospital Dubrava, Zagreb, Croatia; 9 Department of Anatomy, ˝Drago Perovic˝, School of Medicine, University of Zagreb, Zagreb, Croatia; Institute for Clinical and Experimental Medicine, CZECH REPUBLIC

## Abstract

**Introduction:**

Portal hypertension (PH) drives the progression of liver cirrhosis to decompensation and death. Hepatic venous pressure gradient (HVPG) measurement is the standard of PH quantification, and HVPG≥10 mmHg defines clinically significant PH (CSPH). We performed proteomics-based serum profiling to search for a proteomic signature of CSPH in patients with compensated advanced chronic liver disease (cACLD).

**Materials and methods:**

Consecutive patients with histologically confirmed cACLD and results of HVPG measurements were prospectively included. Serum samples were pooled according to the presence/absence of CSPH and analysed by liquid chromatography-mass spectrometry. Gene set enrichment analysis was performed, followed by comprehensive literature review for proteins identified with the most striking difference between the groups.

**Results:**

We included 48 patients (30 with, and 18 without CSPH). Protein CD44, involved in the inflammatory response, vascular endothelial growth factor C (VEGF-C) and lymphatic vessel endothelial hyaluronan receptor-1 (LYVE-1), both involved in lymphangiogenesis were found solely in the CSPH group. Although identified in both groups, proteins involved in neutrophil extracellular traps (NET) formation, as well as tenascin C, autotaxin and nephronectin which mediate vascular contractility and lymphangiogenesis were more abundant in CSPH.

**Discussion and conclusion:**

We propose that altered inflammatory response, including NET formation, vascular contractility and formation of new lymph vessels are key steps in PH development. Proteins such as CD44, VEGF-C, LYVE-1, tenascin C, Plasminogen activator inhibitor 1, Nephronectin, Bactericidal permeability-increasing protein, Autotaxin, Myeloperoxidase and a disintegrin and metalloproteinase with thrombospondin motifs-like protein 4 might be considered for further validation as potential therapeutic targets and candidate biomarkers of CSPH in cACLD.

## Introduction

Chronic liver injury caused by various agents leads to tissue scarring, progressive impairment of liver function and development of portal hypertension (PH), as typically seen in liver cirrhosis. According to the latest data liver cirrhosis accounted for 1.32 million of deaths worldwide, being the 7^th^ cause of disability-adjusted life years among people aged 50–74 years in 2019 [1]. In the initial phase of “compensated cirrhosis” there are no, or only few unspecific symptoms, so the disease might remain unrecognized and progress to liver decompensation, marked by liver dysfunction and/or presence of complications related to PH, such as ascites, variceal bleeding, and hepatic encephalopathy [2]. There is a clear correlation between the severity of PH and the risk of developing these complications, including the risk of dying. Therefore, it is of clinical relevance to precisely assess the grade of PH in order to stratify the prognostic risks, and to adjust the patient’s management accordingly. The gold-standard for quantifying the severity of PH is hepatic venous pressure gradient (HVPG) measurement, which is accomplished through catheterization of hepatic veins. Portohypertensive complications in patients with compensated advanced liver disease (cACLD) start to develop when HVPG exceeds 10 mmHg, which is thus considered the threshold to define clinically significant portal hypertension (CSPH) [3]. Non-selective beta blockers (NSBB) can prevent liver decompensation if introduced to patients with CSPH, even if signs of CSPH are lacking (such as oesophageal varices, or other porto-systemic collaterals) [4]. HVPG measurement is therefore needed for assessing the severity of PH in patients with chronic liver disease (CLD), guiding the decision to initiate NSBB therapy, as well as for assessing the hemodynamic response to treatment. As HVPG measurement is invasive, expensive, and not widely available, there is a need for simple, non-invasive and reliable substitute. The most widely used non-invasive method for this purpose is elastography, especially transient elastography (TE) in combination with the platelet count, and this approach has been endorsed by the Baveno consensus [3]. However, adequate liver stiffness measurements (LSM) depend on the experience of the operator and the constitution of the patient, and might be unreliable in the presence of ascites, liver inflammation or congestion, biliary stasis or infiltrative liver disease [5]. Even if none of the interfering factors hinder accurate diagnosis, in 30–40% of patients it is still not possible to confirm or exclude the presence of CSPH by using LSM and platelet count [6]. Serum biomarkers represent a potential solution to these limitations. Not only could proteomic profiling assist in identifying serum biomarkers for CSPH, but also in profiling related to the hemodynamic response to NSBB administration, which has not been possible with other non-invasive methods so far. In previous studies investigated molecules were chosen based on their known or presumed role in the pathogenesis of PH, but to date none have provided a suitable biomarker superior to TE. As an example, VITRO score (Von Willebrand factor-Ag/thrombocyte ratio) was significantly higher in patients with CSPH, yet its diagnostic accuracy failed to surpass TE [7]. In contrast to the study of individual biomolecules, there has been a growing interest in using omics-based approach to encompass wider molecular clusters involved in the pathophysiology of PH. Proteomics in hepatology was thus far mostly employed to identify biomarkers of fibrosis and inflammation, and a recent study provided a biomarker panel that could successfully detect significant fibrosis, future liver-related events and all-cause mortality in patients with alcohol-related liver disease [8]. However, the pathogenesis of PH is much more complex, and understanding it solely as a consequence of the proliferation of connective tissue in the liver would be overly simplified. Besides fibrosis, as the static component of increased intrahepatic resistance, contraction of hepatic stellate cells (HSC) upon their activation caused by the liver injury, dysfunction of the liver sinusoidal endothelial cells (LSEC), microvascular thrombosis and neoangiogensis represent the dynamic component of PH and contribute together to the altered intrahepatic haemodynamic conditions that furtherly aggravate PH and liver injury [9]. As the result of the intestinal congestion and increased permeability bacterial translocation develops and triggers inflammatory response, nitric oxide mediated splanchnic vasodilatation, and further intrahepatic endothelial dysfunction. Splanchnic vasodilatation leads to increased portal inflow and subsequent increased portal pressure [10]. Thus, the wider approach is obviously needed in the quest for identifying potential biomarkers of PH, yet proteomic studies in this lane are scarce. Previous studies mostly used inadequate models that hindered the translation of their results to the real-world clinical practice. One study used proteomic profiling of gastric mucosa samples from patients with endoscopic finding of portal gastropathy, and healthy individuals, and they identified 17 differently expressed proteins in the portal gastropathy group, with the most prominent being tenascin-C (TN-C) and fibulin-5. These proteins relate to angiogenesis and lymphangiogenesis, as the adaptive and compensatory mechanisms activated along the PH development [9,11,12]. Osborn et al. have compared the serum proteomic profiles of children with biliary atresia and identified five protein candidates (SEMA6B, SFRP3, COMMD7, BMX, and VCAM1) in children with clinical signs of CSPH [13]. Their presumed role in the pathogenesis of PH might be illustrated by the Bone marrow kinase on chromosome X (BMX) protein that is involved in the regulation of proinflammatory cytokines secretion [14], whereas Vascular cell adhesion molecule 1 (VCAM-1) is involved in endothelial activation and angiogenesis [15]. However, in both these studies PH was diagnosed using clinical criteria and not HVPG measurement, thus failing to include patients who might already had CSPH, but without overt clinical manifestation, which is exactly the most challenging group for early diagnosis of CSPH. In addition, biliary atresia refers to the specific population, and the results might not be extrapolated to an adult population with a different aetiology of liver disease. The most recent study demonstrated a diminished transcription of chromobox 7, as a pressure-sensitive transcription factor in primary cultured LSEC exposed to increased hydrostatic pressure, which led to increased plasma levels of E-cadherin and serine protease inhibitor Kazal-type 1 in patients with ACLD and PH. Consequently, these two proteins have been proposed as potential biomarkers of PH and CSPH, but need further validation in a larger cohort of well dissected patients with cACLD with and without CSPH [16].

Timely intervention in cACLD patients with CSPH can alter the course of their disease, making an early diagnosis of CSPH crucial. The best candidate for this should be easily obtained, widely available, cheap and reliable, pointing to the blood-derived test as probably the most suitable. Therefore, the primary aim of this pilot study was to search for proteomic signature of CSPH in patients with cACLD which might reveal candidate proteins to be furtherly evaluated as the potential biomarkers of CSPH. Secondary aim was to get further insights into the transition from subclinical PH to CSPH in patients with cACLD based on differential presence of some proteins in these two stages of PH development, which might represent potential targets for further therapeutic interventions.

## Materials and methods

### Study participants and study outline

**Fig 1:**
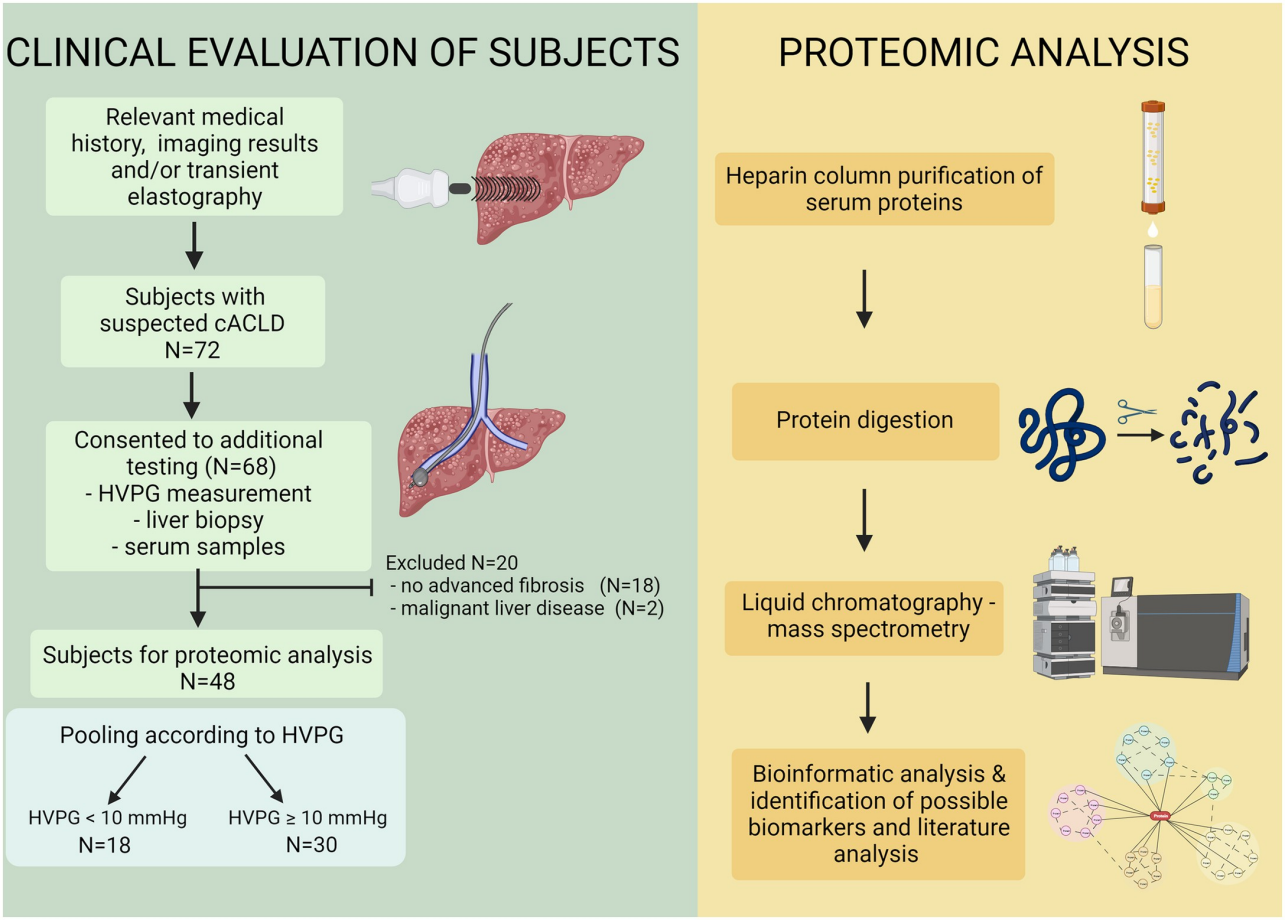
Study outline showing the subject groups and the methodological approach. Proteomic data were obtained from the pooled and purified serum samples of patients with compensated advanced chronic liver disease (cACLD), divided in two groups related to the presence of clinically significant portal hypertension. Created with BioRender.com. Abbreviations: cACLD = compensated advanced chronic liver disease; HVPG–Hepatic venous pressure gradient.

This cross-sectional observational study was preformed from 1^st^ of July 2018 till 1^st^ of March of 2020, and it included consecutive patients with a history of chronic liver disease and a suspicion of cACLD based on the results of imaging and/or LSM ≥10 kPa as measured by TE (Fibroscan^®^, Echosens, France), who were referred to HVPG measurement and transjugular liver biopsy. Patients below 18 years of age, those with current or previous liver decompensations or those allergic to iodine contrast agents were also excluded. Seventy-two patients suspected of having cACLD were invited and 68 provided consent to participate in the study. Aetiology of liver diseases was defined based on history data of alcohol abuse (AUDIT-C score ≥3 for women and ≥4 for men; on-line calculator, available at https://www.mdcalc.com/calc/2021/audit-c-alcohol-use), significant exposure to hepato-toxic drugs, presence of metabolic syndrome, and standardized laboratory and imaging work-up used to confirm/exclude chronic viral hepatitis, autoimmune-associated liver diseases and metabolic liver diseases (such as Wilson’s disease and hereditary haemochromatosis). From the initial cohort of 68 patients, excluded were those without histological evidence of advanced fibrosis (N = 18) because CSPH is very rare in earlier stages of liver fibrosis. We also excluded 2 patients with liver malignancy as revealed on the further imaging studies (Fig 1). The final cohort comprised 48 patients with histologically confirmed advanced fibrosis/cirrhosis and available results of HVPG measurements. A 5 mL of blood was acquired from each subject and stored for proteomic analysis. The study was approved by the Institutional Ethics committee and all patients provided written informed consent.

### HVPG measurement and transjugular liver biopsy

HVPG measurement was performed using a transjugular approach by catheterization of the right hepatic vein. A venous introducer (Arrow 45 cm/7 Fr; Kimal CL-07745) was placed and under fluoroscopic control, a balloon-tipped occlusion catheter (Berenstein Catheter,Boston S, ref. M001173010) was introduced in the hepatic vein. The difference between wedged hepatic vein pressure and free hepatic vein pressure was measured in triplicate and the mean value was calculated for further analysis [17]. During the same procedure, with introducer sheet in right hepatic vein, trucut needle (Bio-Cut semi-automatic biopsy device 19 G/60 cm, Kimal) was introduced and liver biopsy was performed. Three samples of liver tissue were obtained for pathohistological analysis, and one additional if needed for determining Cu or Fe in the dried tissue, as clinically indicated. All biopsy samples were paraffin-embedded, sliced, stained by Hematoxylin-Eosin and Masson trichrome, and analysed by a single in-house experienced hepato-pathologist, who was blinded of clinical data. The presence of cACLD was considered in biopsies demonstrating bridging fibrosis or cirrhosis, as per Ishak classification [18]. All patients included in the final cohort (N = 48) had both liver biopsy and HVPG measurements available.

### Serum sample collection and heparin column chromatography

Blood samples were acquired by venipuncture in tubes without anticoagulants and serum was obtained by centrifugation (15 min at 3000 *g*) and aliquots of each sample were stored at –80 ºC until analysis. In the next step samples were pooled based on the CSPH into two groups: 1-patients with CSPH (N = 30), and 2-patients without CSPH (N = 18) [19].

To reduce the abundance of albumins and immunoglobulins, and to allow for the analysis of less represented proteins that are involved in biological processes pooled samples were purified on the heparin sepharose affinity column (HiTrap Heparin HP, Thermo Fisher Scientific, Waltham, MA, USA) two times and eluted with 5 mL of sodium chloride solution in three fractions (0.5, 1 and 2 M) [20].

Proteins from the eluates were then precipitated with cold acetone and then left overnight to precipitate at -80 ºC. The following day, pooled samples were centrifuged (10 min at 16,000 *g*), supernatants removed, and the protein pellets resuspended with phosphate-buffered saline and stored at -80 ºC until further analysis.

### Liquid Chromatography-Mass Spectrometry (LC-MS) analysis

Protein concentrations were determined spectrophotometrically using the Lowry protein assay (BioRad, Hercules, CA, USA) according to the manufacturer’s instructions. Protein digestion was performed in 10 kDa molecular weight cut-off spin filters, according to standard protocols. Briefly, proteins samples (40 μg) were denatured (8 M urea), alkylated (55 mM iodacetamide in 8 M urea) and digested with 0,8 μg of TPCK treated trypsin (Worthington Industries, Columbus, OH, SAD). Tryptic peptides were finally concentrated and purified using C18 StageTips columns [21]. Prior to mass spectrometry (MS), peptides were separated in a gradient of acetonitrile in formic acid on a PepMap C18 25-centimetre-long-nano-column by high-performance-liquid-chromatography on an Easy-nLC 1200 System (Thermo Fisher Scientific, Waltham, MA, USA). Mass spectrometry was performed on a Q Exactive Plus instrument (Thermo Fisher Scientific, Waltham, MA, USA). Automated measurement cycles consisted of full MS scanning and MS/MS scanning of up to ten most intense ions. Full MS scans ranging from m/z 300 to 1800, were obtained in the Orbitrap analyser at a resolution of 70,000, with internal calibration of the instrument using the lock mass setting. The MS raw data were deposited at the ProteomeXchange Consortium via the PRIDE partner repository and are available via ProteomeXchange with identifier PXD040807.

### Data analysis

Raw data was processed using the Proteome Discoverer (Thermo Fisher Scientific, Waltham, MA, USA) software version 2.4. Spectral data was searched using SequestHT and Mascot search engines in iterative manner against human proteome database retrieved from UniProt (Release 2020_05, Proteome ID: UP000005640). The search parameters were full trypsin digest with a maximum of two missed cleavages allowed. Dynamic modifications were: Met oxidation, Asn and Gln deamination. Dynamic protein N-terminal modifications: Met-loss, and acetylation. Common laboratory contaminants and proteins with a false discovery rate over 1%, were not included in the analysis. Abundances of individual proteins were determined using the label-free quantification (LFQ) algorithm to obtain intensity values that are proportional to the molar quantities of individual proteins. LFQ values were calculated from MS1 peptide intensities and normalized between samples. Samples were analysed in technical triplicates, and proteins identified with at least one peptide were considered relevant for analysis.

The normality of distribution for patients’ characteristics was tested informally by plotting histograms and formally by using Kolmogorov-Smirnov test. Since the numerical variables were not normally distributed, they are presented as median accompanied by 1^st^ and 3^rd^ quartile values and compared using the Kruskal-Wallis test. Categorical variables are presented with the absolute number and a percentage and were compered between groups using chi-square (X^2^) test. Statistical analysis was performed using MedCalc statistical software version 22.001 (MedCalc Software Ltd, Ostend, Belgium).

Gene set enrichment analysis of the acquired data was performed using Funrich 3.1.3 software. The analysis included determining the involved biological processes of the most biologically relevant abundant proteins [22]. The software employed the hypergeometric test, corrected for multiple comparisons by Bonferroni method, to evaluate statistical significance of analysed parameters, such as relevant biological processes. Type one error (alpha) was set to 0.05.

String database version 11.5 was used to analyse the selected protein interaction networks and classify the interactome nodes according to the most relevant gene ontology annotations, functional pathways and tissue localization [23]. For this we have selected proteins exclusively present in CSPH, as well as those present in both groups, but with >2-fold higher intensity in the CSPH group (altogether 82 proteins).

A comprehensive literature review using MEDLINE was performed for proteins that had the most striking difference in abundance among two pools, as well as for the biological processes that were more pronounced in either of groups. Individual proteins pertaining to processes that were deemed to be pathophysiologically relevant in CLD and PH development were chosen by manual curation.

## Results

The final cohort analysed in this study comprised 48 patients with histologically confirmed cACLD, of which 30 patients had CSPH. Demographic and clinical data of these patients are presented in Table 1: men prevailed, and there were no significant differences in terms of age or body mass index between the groups with and without CSPH. The most prevalent aetiology was steatotic liver disease, with most of the patients in both groups having overlap of metabolic dysfunction and history of excessive alcohol consumption [24]. Yet, majority of study participants were in abstinence for >3 months at the time of inclusion into the study and the groups didn’t differ neither regarding the aetiology of the liver disease nor activity of alcohol consumption. As expected, patients with CSPH had higher LSM, AST to platelet ratio index (APRI) and FIB-4 (Fibrosis-4 index), as well as worse biochemical indicators of liver function. All included patients were compensated with numerically low Child-Pugh and MELD scores, yet these scores were significantly higher in patients with CSPH. Database with the individual data of the included participants is available as S1 Table.

**Table 1:** Characteristics of patients involved in the study.

		CSPH (N = 30)	Non-CSPH (N = 18)	p-value
**Demographics**	Age (years)	62 (57–68)	60.5 (56–64)	0.376
	Sex (male)	22 (73.30%)	14 (77.80%)	0.733
	BMI (kg/m^2^)	25.4 (22.6–27.9)	26.84 (25.5–31.4)	0.686
**Aetiology**	MASLD	6 (20%)	5 (27.8%)	0.865
	MetALD	15 (50%)	7 (38.9%)	
	ALD	3 (10%)	1 (5.6%)	
	Viral (HBV/ HCV)	3 (10%)	2 (11.1%)	
	Other	3 (10%)	3 (16.7%)	
	Active alcohol consumption	7 (23.3%)	3 (16.7%)	0.701
**Fibrosis assessment**	LSM (kPa)	34.3 (26.0–53.0)	14.1 (11.6–20.0)	**<0.001**
	Fibrosis stage (Ishak)	6 (6–6)	5.5 (4–6)	**0.012**
**Biochemistry**	Bilirubin (μmol/L)	18 (9–36)	12 (10.8–16.3)	**<0.001**
	Albumin (g/L)	35.5 (32–40)	44 (43–46)	**<0.001**
	INR	1.255 (1–1.47)	1.025 (1–1.08)	**0.046**
	Creatinine (μmol/L)	66.5 (55–82)	69.5 (62–84)	0.717
	Sodium (mmol/L)	138 (137–140)	138.5 (138–140)	0.448
	Platelets (x10^9^/L)	111.5 (77–162)	202.5 (161–255)	**<0.001**
**Calculated scores**	MELD score	11.5 (9–15)	9 (8–11)	**0.004**
	Child Pugh score	5.5 (5–7)	5 (5–5)	**0.006**
	APRI	1.4 (0.85–2.27)	0.545 (0.39–0.65)	**<0.001**
	FIB-4	4.575 (2.85–6.42)	1.965 (1.3–2.48)	**<0.001**
**Hemodynamics**	HVPG (mmHg)	16.2 (12–18)	6 (5–7)	**<0.001**

Abbreviations: CSPH = clinically significant portal hypertension. BMI = body mass index; MASLD = metabolic dysfunction associated steatotic liver disease; MetALD = metabolic dysfunction associated steatotic liver disease and increased alcohol intake; ALD = alcohol-associated liver disease; HBV = hepatitis B virus; HCV = hepatitis C virus; LSM = liver stiffness measurement; INR = international normalized ratio; MELD = model for end stage liver disease; APRI = AST to platelet ratio index; FIB-4 = Fibrosis-4 index; HVPG = hepatic venous pressure gradient.

**Fig 2:**
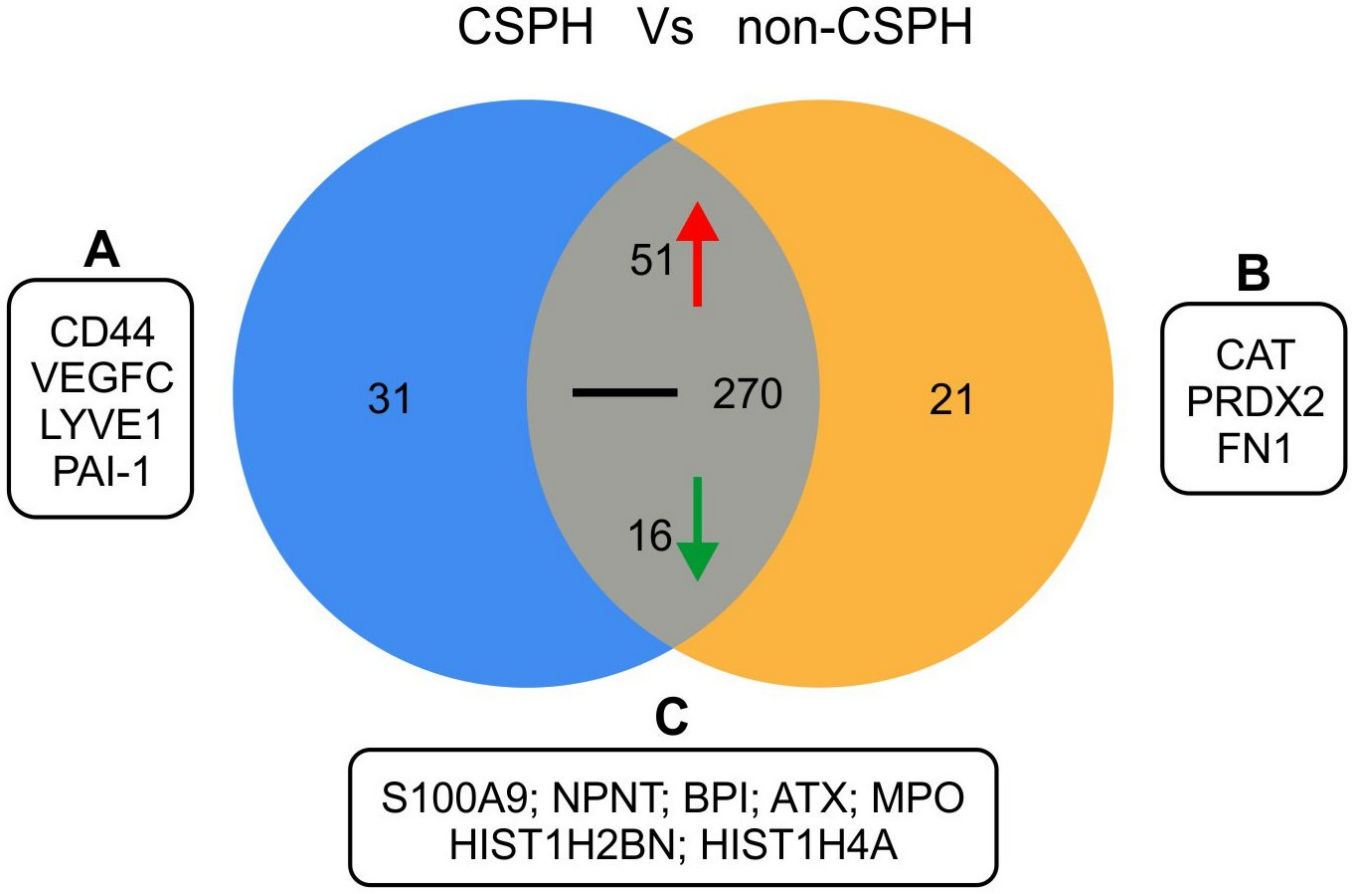
Quantitative Venn diagram of proteins expressed in sera of patients with clinically significant portal hypertension (CSPH) and non-CSPH group. Red and green arrows denote proteins that were respectively upregulated and downregulated in CSPH (> 2-fold increase/decrease in intensity levels), whereas black line depicts no, or less than 2-fold change in intensity. Black boxes highlight identified proteins with plausible pathophysiological role in PH development among those identified (A) solely in CSPH group, (B) solely in non-CSPH group, and (C) represented in both groups but with >2-fold higher intensity in CSPH group. Abbreviations: ATX–Autotaxin; BPI–Bactericidal permeability increasing protein; CAT–Catalase; CD44 –Cluster of differentiation 44; FN1 –Fibronectin; HIST1H2BN–Histone H2B; HIST1H4A –Histone H4; LYVE1 –Lymphatic vessel endothelial hyaluronic acid receptor 1; MPO–Myeloperoxidase; NPNT–Nephronectin; PAI-1 –Plasminogen activator inhibitor 1; PRDX2 –Peroxiredoxin-2; S100-A9—Protein S100-A9.

Following affinity purification of the sera on a heparin column, a total of 389 proteins were identified by LC-MS, 368 in the CSPH group and 358 in the group without CSPH (S2 Table). Out of total 389 proteins, 31 were identified solely in the CSPH group and 21 only in non-CSPH group **(**Fig 2 and S3 Table). Analysis of these outliers revealed several proteins with the plausible role in the pathogenesis of PH, as highlighted in Table 2. Among the proteins identified solely in the CSPH group, the most interesting findings were the abundance of Cluster of differentiation 44 (CD44), involved in the inflammatory response, plasminogen activator inhibitor 1 (PAI-1) that facilitates clot formation, as well as vascular endothelial growth factor C (VEGF-C) and lymphatic vessel endothelial hyaluronan receptor-1 (LYVE-1), which have roles in the formation of lymphatic vessels [25–28]. Among the proteins detected in non-CSPH groups, peroxiredoxin and catalase are known for their protective activity against oxidative stress, while fibronectin is important for extracellular matrix (ECM) assembly and bacterial opsonisation [29–31]. For other proteins exclusively identified in each group, as listed in S3 Table, we were not able to find reasonable association with the progression of liver disease and development of PH based on the available data in the literature.

**Table 2:** Proteins identified as potential candidate biomarkers for CSPH. Proteins selected by manual curation based on their plausible role in the pathogenesis of portal hypertension.

**Proteins identified only in the CSPH group**			
**Protein**	**Role**		**Pathogenetic pathway**
Cluster of differentiation 44 antigen (CD44)	Mediates inflammatory response by inducing the release of proinflammatory cytokines, and migration of macrophages and neutrophils [25,32]		Inflammation
Vascular endothelial growth factor C (VEGFC)	Increased levels during the repair phase of ischemia reperfusion liver injury. Induces lymphangiogenesis and has proangiogenic activity [12,28]		Angiogenesis, lymphangiogenesis
Lymphatic vessel endothelial hyaluronic acid receptor 1 (LYVE1)	Receptor for hyaluronic acid and mediator of lymphangiogenesis [27,33]		Lymphangiogenesis
Plasminogen activator inhibitor 1 (PAI-1)	Promotes thrombotic risk and can aggravate liver disease by local ischemia [26,34]		Coagulation
**Proteins identified only in the non-CSPH group**			
**Protein**	**Role**		**Pathogenetic pathway**
Catalase	Degrades hydrogen peroxide. Antioxidant [30]		Oxidative stress
Fibronectin	Plays a role in cellular adhesion and migration, wound healing, phagocytosis, cell matrix assembly, and opsonisation of bacteria [31,35]		Cell adhesion, immune response
Peroxiredoxin-2	Eliminates peroxides generated during metabolism together with catalase and glutathione peroxidase [29]		Oxidative stress
**Proteins identified in both groups, with > 2-fold increased intensity in CSPH group**			
**Protein**	**Fold increase in CSPH group**	**Role**	**Pathogenetic pathway**
Protein S100-A8 and A9	22.60	Involved in NET formation [36]	Inflammation
Histone H1.5	16.18	Involved in NET formation [37]	Inflammation
Histone H1.4;Histone H1.3	6.49	Involved in NET formation [37]	Inflammation
Tenascin C (TN-C)	5.80	Inflammation and fibrinogenesis, upregulated in lymphatic endothelial cells during lymphangiogenesis [38–40]	Inflammation, HSC activation, lymphangiogenesis
Nephronectin (NPNT)	3.84	Promotes infiltration of CD4+ T cells or NKTs. Enhances contraction of HSC [41,42]	Inflammation, HSC activation
Bactericidal permeability-increasing protein (BPI)	3.53	Involved in NET formation, bactericidal properties [37,43]	Inflammation
A disintegrin and metalloproteinase with thrombospondin motifs-like protein 4 (ADAMTSL4)	3.46	Involved in ECM organization by inducing fibrillin-1 deposition, and increased fibrilin-1 deposition is present in cirrhosis [44,45]	Fibrogenesis
Autotaxin (ATX) (Ectonucleotide pyrophosphatase/phosphodiesterase family member 2)	3.12	Smooth muscle contraction, platelet aggregation, angiogenesis, cell migration, as well as stimulation of proliferation and contractility of HSC [46,47]	Coagulation, angiogenesis, HSC activation
Myeloperoxidase (MPO)	2.45	Involved in NET formation. Bactericidal properties. Contributes to tissue damage at sites of inflammation. Activates HSCs and upregulates TGF-β [48,49]	Inflammation, HSC activation
**Proteins identified in both groups, with > 2-fold decreased intensity in CSPH group**			
**Protein**	**Fold decrease in CSPH group**	**Role**	**Pathogenetic pathway**
Junction plakoglobin (JUP)	18.60	Maintains endothelial cells junction rigidity and regulates membrane permeability[50]	Cell adhesion
Syntaxin-binding protein 5 (STXBP5)	3.64	Regulator of endothelial exocytosis [51]	Coagulation
Annexin A2 (ANXA2)	3.01	Underexpression is linked to thrombosis. Involved in thrombosis, inflammation and has a role in response to tissue injury and can participate in pathogenic response leading to tissue fibrosis [52]	Coagulation, inflammation, fibrogenesis

Abbreviations: CSPH = clinically significant portal hypertension; NET = neutrophile extracellular traps; NKTs = natural killer T cells; HSC = hepatic stellate cells; TGF-β = Transforming growth factor beta.

**Fig 3:**
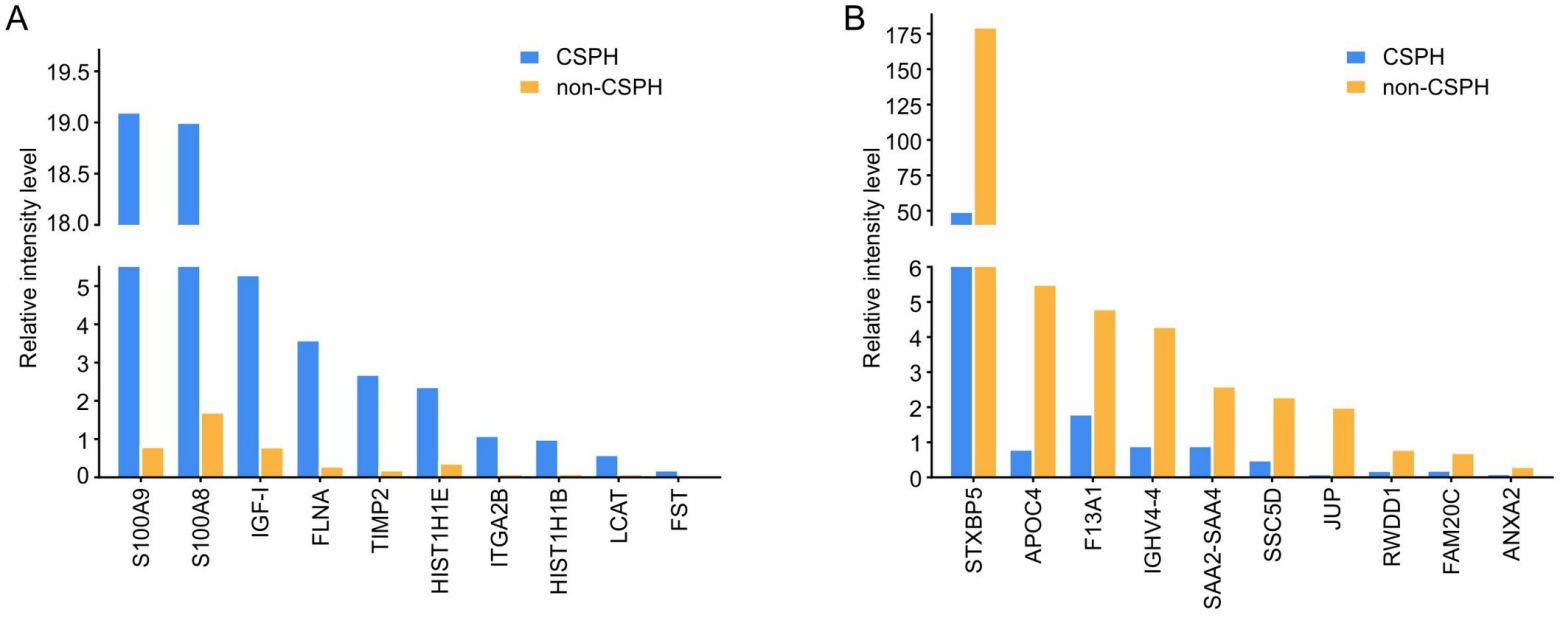
Proteins present both in patients with CSPH and no CSPH, identified with the highest fold-increase in their relative intensity levels in the sera of CSPH (A) and non-CSPH patients (B). Abbreviations: ANXA2 –Annexin A2; APOC4 –Apolipoprotein C-IV; FLNA–Filamin-A; HIST1H1B –Histone H1.5; HIST1H1E –Histone H1.4;Histone H1.3; IGF-I–Insulin-like growth factor 1; IGHV4-4 –Immunoglobulin heavy variable 4–4; ITGA2B –Integrin alpha-Iib; JUP–Junction plakoglobin; LCAT–Phosphatidylcholine-sterol acyltransferase; F13A1 –Coagulation factor XIII A chain; FAM20C –Extracellular serine/threonine protein kinase; FST–Follistatin; RWDD1 –RWD domain-containing protein 1; S100-A8 –Protein S100-A8; S100-A9 –Protein S100-A9; SAA2-SAA4 –Serum amyloid A-4 protein; SSC5D –Soluble scavenger receptor cysteine-rich domain-containing protein; STXBP5 –Syntaxin-binding protein 5.

Among the 337 proteins identified in both groups (overlap), 51 proteins had > 2-fold intensity increase, and 16 proteins had > 2-fold intensity decrease in the CSPH group compared to the non-CSPH group, whereas remaining 270 proteins had no, or < 2-fold difference in intensity between the groups (Fig 2). Of these 337 overlapping proteins, those with the most striking difference in intensity between the groups are depicted in Fig 3, whereas all proteins with>2-fold difference in intensity (in positive or negative direction) for both groups are listed in S3 Table. Several of these proteins are highlighted as they have been associated to the progression of liver disease and PH. The most significantly abundant proteins in CSPH group were those involved in neutrophile extracellular traps (NET) formation such as S100-A8, S100-A9, histones H1, H2B and H4, whereas Filamin-A, Insulin-like growth factor 1 and Metalloproteinase inhibitor 2 mediate liver inflammatory response and tissue remodelling. Other proteins with significant abundance in CSPH were Bactericidal permeability-increasing protein (BPI), TN-C, autotaxin (ATX) and nephronectin (NPNT), pointing to the important role of gut bacteria, vascular contractility and lymphangiogenesis along the worsening of cACLD and development of PH (Table 2).

**Fig 4:**
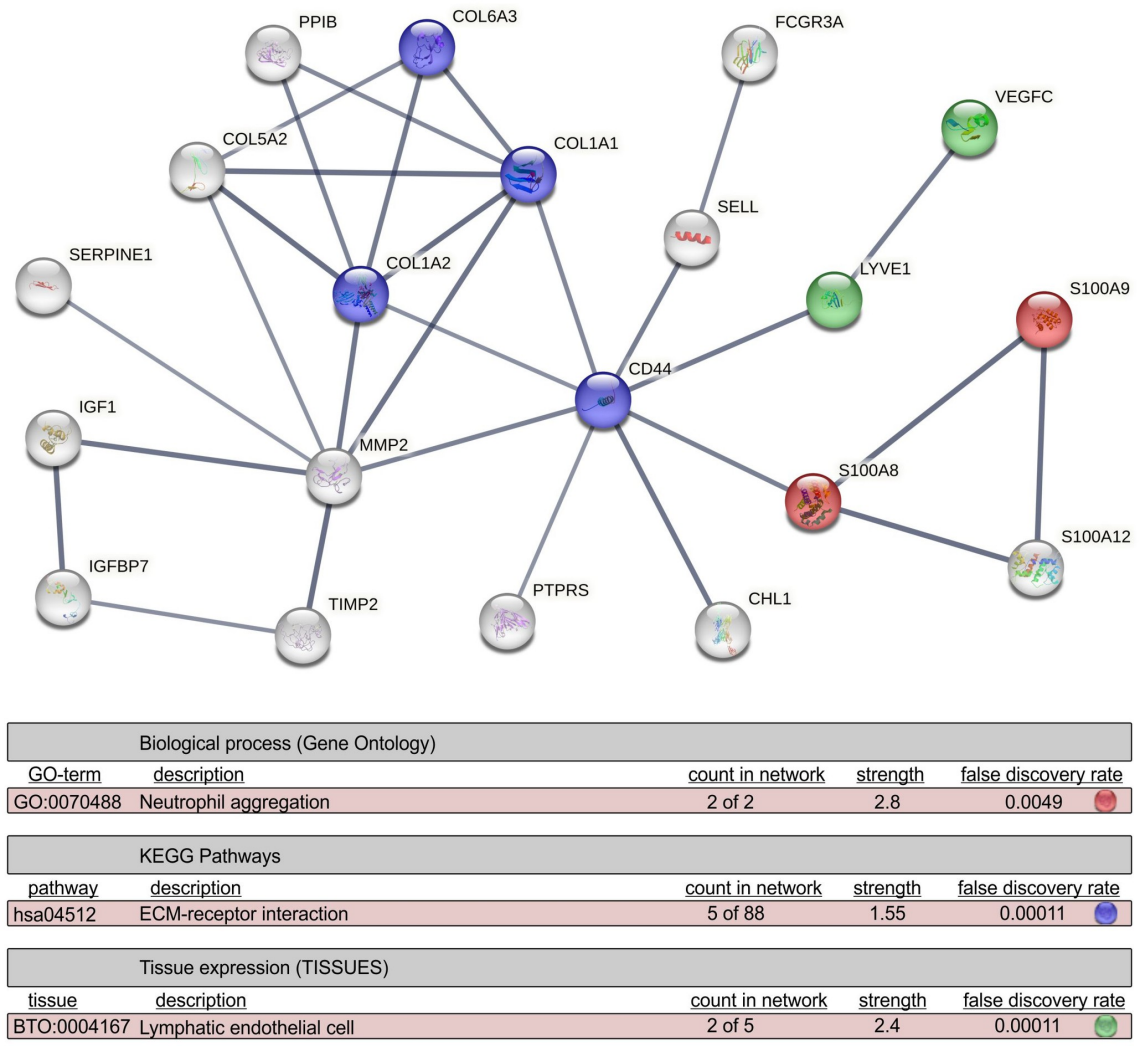
Interaction network of protein cluster identified exclusively in clinically significant portal hypertension (CSPH), and proteins >2-fold upregulated in sera of CSPH *vs* non-CSPH patients. Line thickness indicates the strength of data support. Proteins with interaction score under 0.7 (designating low and medium confidence interactions) and disconnected nodes are excluded from the network. Functional enrichment analysis reveals the most significant (i.e. with the highest strength and lowest false discovery rate) biological process (red nodes), KEGG pathways (blue nodes) and tissue expression patterns (green nodes) of the depicted proteins. Abbreviations: CD44 –Cluster of differentiation 44; CHL1 –Close homolog of L1; COL-(1A1,1A2, 5A2, 6A3)–Collagen subtypes; FCGR3A –Low affinity immunoglobulin gamma Fc region receptor III-A; IGF-I–Insulin-like growth factor 1; IGFBP7 –Insulin-like growth factor-binding protein 7; KEGG–Kyoto Encyclopedia of Genes and Genomes; LYVE1 –Lymphatic vessel endothelial hyaluronic acid receptor 1; MMP2–72 kDa type IV collagenase; PPIB–Peptidyl-prolyl cis-trans isomerase B; PTPRS–Receptor-type tyrosine-protein phosphatase S; S100-(A8, A9, A12)–Protein S100 –(A8, A9, A12); SELL–L-selectin; SERPINE1 –Plasminogen activator inhibitor 1.

Interactome analysis revealed significant clustering of proteins identified exclusively in CSPH or upregulated in sera of patients with CSPH. Functional enrichment analysis also indicated inflammation, ECM-receptor interaction and lymphangiogenesis as important pathogenetic mechanisms involved in progression from subclinical PH to CSPH (Fig 4).

**Fig 5:**
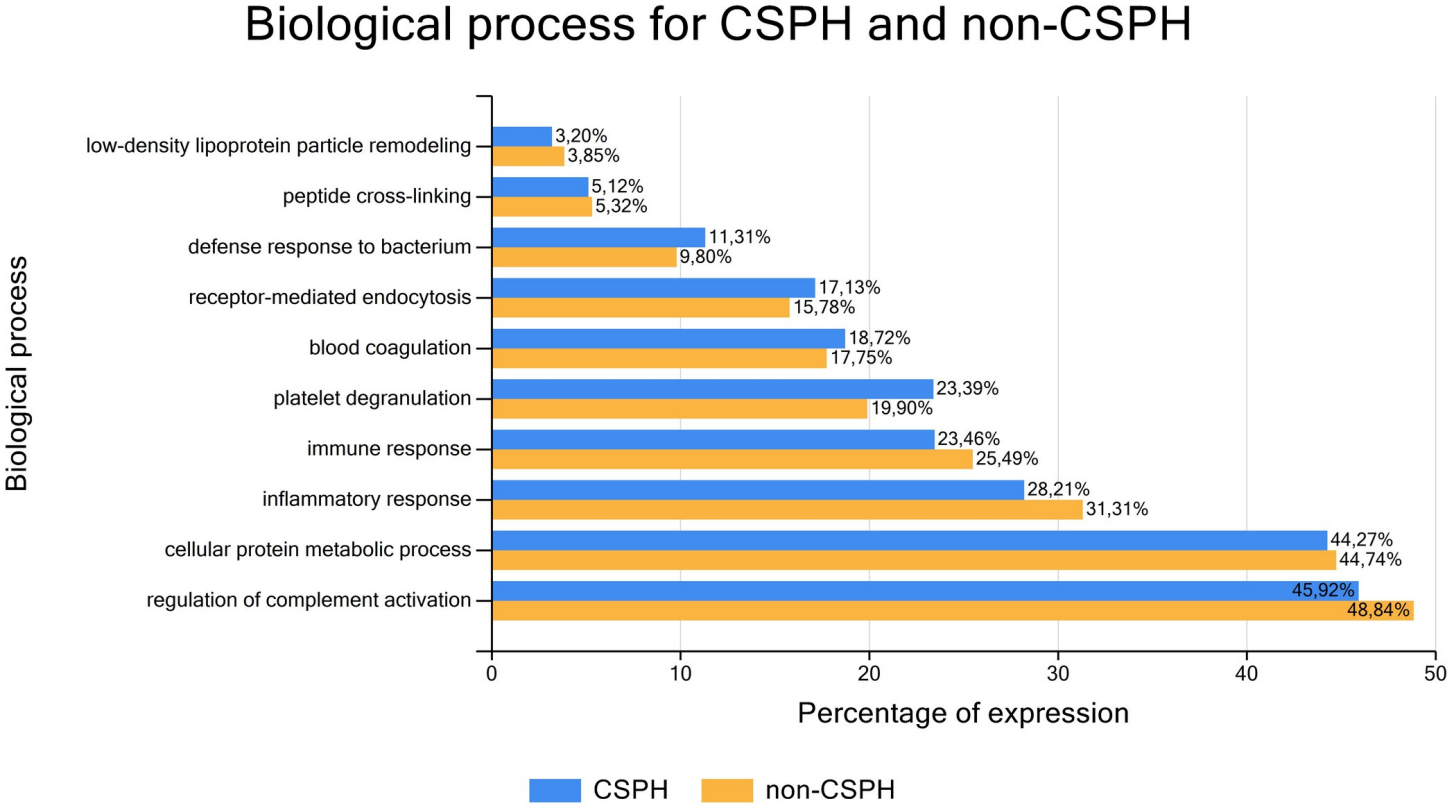
Functional enrichment depicting the biological processes associated to the proteins detected in the sera of clinically significant portal hypertension (CSPH) and non-CSPH patients, listed from smallest to largest ratio (percentage) of proteins related to the respective process. X-axis: Percentage of detected proteins associated to a specific biological process, Y-axis: Biological process identified by functional enrichment. Abbreviations: CSPH = clinically significant portal hypertension.

Functional enrichment analysis yielded 40 statistically significant biological processes represented in both groups of severity of PH. Interestingly, processes pertaining all aspects of inflammatory response, including complement activation, regulation of immune response, blood cells degranulation, phagocytosis and engulfment, as well as defence response to bacterium prevailed by far, underlying again importance of gut-liver crosstalk in the pathogenesis of CLD and PH development. The representative biological processes identified with statistical significance in patients with and without CSPH are depicted in Fig 5, and a complete list of all 40 significant biological processes in S1 Fig.

## Discussion

In the present pilot study, we have shown distinctive proteomic signature of CSPH in patients with cACLD. To our knowledge, this is the first study in humans to address this issue narrowed specifically to this stage of CLD. This is of huge clinical importance because exactly at the cACLD stage PH develops in most cases, and then progresses to become the driving force of further deterioration of liver function, development of complications related to PH, and adverse outcomes. For this reason, it is important to recognize CSPH at the stage with no overt clinical signs, as well as to understand the mechanisms underlying the transition from subclinical PH to CSPH, because at this point focused therapeutic intervention might stop or delay the detrimental progression of PH and worsening liver function.

Our results demonstrate prevailing presence of inflammatory and immune-mediated processes, pathways related to response to microbial agents, interconnected with the activation of coagulation, fibrogenesis and the development of neo-angiogenesis and lymphangiogenesis, as the pathogenetic landscape of this stage of CLD. At the individual level proteins such as CD44, PAI-1, VEGF-C, LYVE-1 were exclusively identified in CSPH, whereas abundant presence of proteins involved in NET formation (such as myeloperoxidase (MPO), proteins S100-A8 and S100-A9, histones H2B and H4 and BPI) was demonstrated in CSPH group as compared to non-CSPH group [36,37].

Disturbance of gut-liver axis is an important step in the pathogenesis of PH, characterized by the increased intestinal barrier permeability, bacterial translocation and influx of bacterial products to the liver. This also triggers mesenteric and liver inflammatory response, perpetuating liver damage, and splanchnic vasodilatation, all of which drive the rise in portal pressure [53]. NETs are released from the neutrophils to fend off pathogens in the blood stream. They have also been proposed to propagate inflammation and to play a role in thrombus formation and subsequent parenchymal extinction [48,54]. The NETs are mostly composed of DNA and histones, but also neutrophil-resident proteins, such as neutrophil elastase, MPO and cathepsin G [36,37], and we have identified abundant presence of proteins involved in NET formation (including MPO, proteins S100-A8 and S100-A9, histones H2B and H4, and BPI) in CSPH group as compared to non-CSPH group. MPO, which was increased in CSPH group, is secreted by neutrophils, monocytes, macrophages and Kupffer cells, exerts bactericidal properties, but also contributes to tissue damage, activates HSCs and upregulates TGF-β. In previous studies higher serum levels of MPO were associated with NET formation, severe liver dysfunction, and symptoms of PH [48,49]. This suggests that MPO plays a role both in the pathogenesis of CSPH and a potential protective role against the increased influx of bacteria and their products. Increased expression of BPI in the CSPH group, is in line with findings from the studies that demonstrated a steady increase of BPI along the worsening stages of liver cirrhosis, probably as a result of increasing endotoxin translocation [43]. In the non-CSPH group we exclusively identified peroxiredoxin and catalase, proteins known to protect against oxidative stress, as well as opsonin activity against bacteria. Aller et al. have shown decreased levels of catalase in the liver of rats with cirrhosis and PH compared to those with cirrhosis alone [30]. This suggests that the protective, anti-oxidative effect of catalase is more preserved in non-CSPH patients and might decrease as PH progresses.

**Fig 6:**
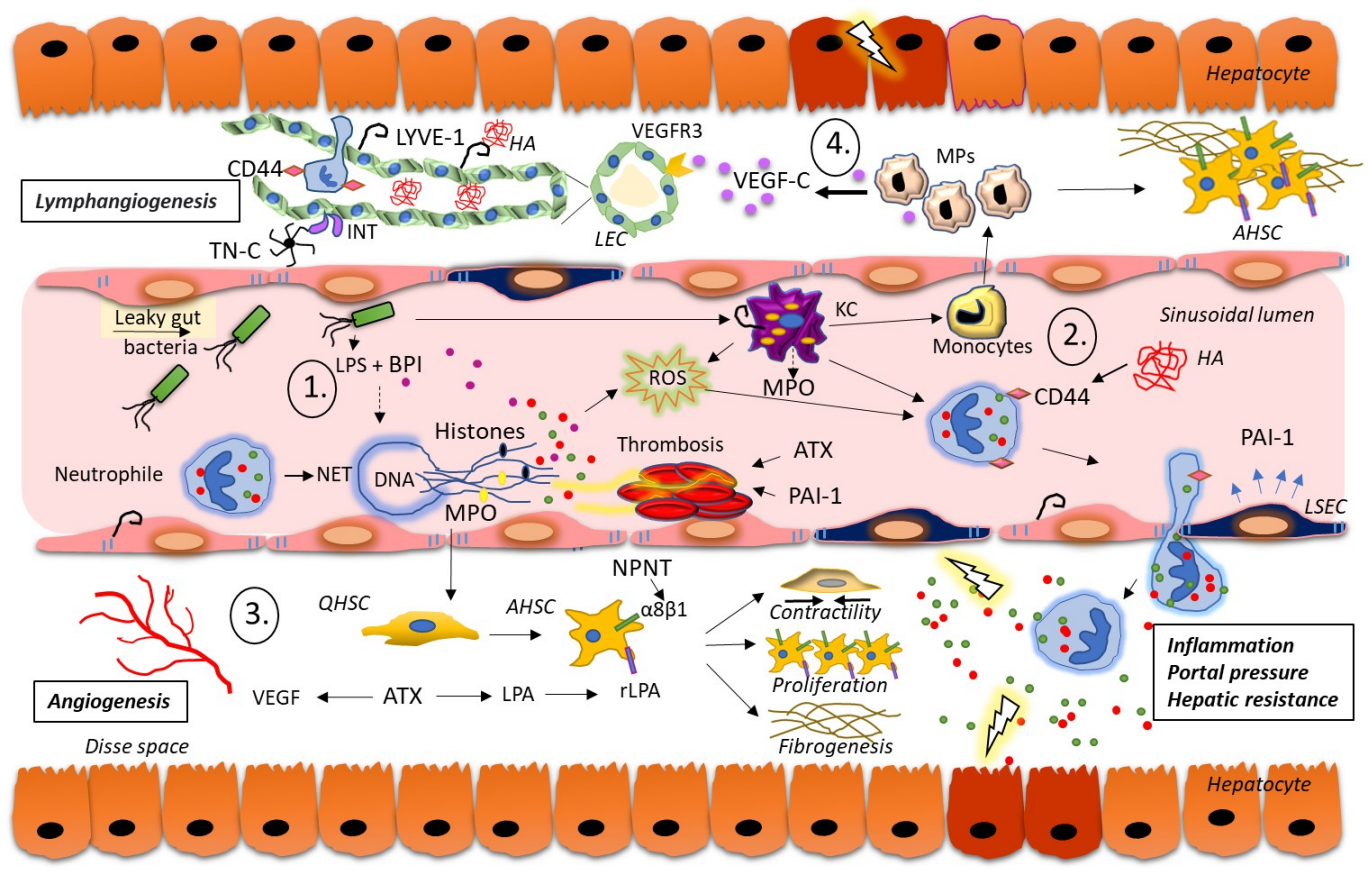
Overview of the proteins identified in patients with CSPH and their proposed role in pathogenesis and progression of PH in cACLD. **1.** Gut bacterial products, immune complexes, and platelets trigger the formation of NET, which promote sinusoidal damage and microthrombosis supported by PAI-1 from activated endothelium. **2.** Damage to LSEC leads to increased HA production which in turn increases CD44 mediated neutrophil sequestration, perpetuates inflammation and HSC activation. **3.** ATX and NPNT influence HSC activation, contractility and fibrogenesis. **4.** VEGF-C from macrophages, LYVE-1, CD44 and TN-C induce lymphangiogensis. Abbreviations: AHSC–activated hepatic stellate cell; ATX–Autotaxin; BPI—Bactericidal permeability increasing protein; cACLD—Compensated advanced chronic liver disease; CD44—Cluster of differentiation 44; CSPH—Clinically significant portal hypertension; HA—Hyaluronic acid; INT–Integrin; KC—Kupffer cells; LEC–lymph vessel endothelial cell; LPA- Lysophosphatidic acid; LPS–Lipopolysaccharide; LYVE1—Lymphatic vessel endothelial hyaluronic acid receptor 1; MP–Macrophage; MPO–Myeloperoxidase; NET–Neutrophil extracellular traps; NPNT–Nephronectin; PAI-1—Plasminogen activator inhibitor 1; PH—Portal hypertension; QHSC–quiescent hepatic stellate cell; rLPA—Lysophosphatidic acid receptor; ROS—Reactive oxygen species; TN-C—Tenascin C; VEGFR3 –Vascular endothelial growth factor receptor 3.

Apart from the injury to liver parenchymal cells mediated by direct toxic effects or immune-mediated mechanisms, significant changes in liver microvasculature can be observed in the development of cirrhosis and PH. HSCs and LSEC are important cellular mediators of the increased intrahepatic vascular resistance. HSCs have drawn attention, as their perisinusoidal contraction in liver injury is perceived as a compensatory mechanism for blood flow regulation [9]. Accordingly, in the CSPH group, we have detected the upregulation of ATX and NPNT proteins known to induce the contractility of HSC. ATX is a secreted enzyme important for smooth muscle contraction, platelet aggregation, angiogenesis, cell migration, and stimulation of proliferation and contractility of HSC [47]. In line with our data, serum ATX demonstrated better diagnostic performance for hepatic encephalopathy and variceal rupture in patients with cirrhosis in comparison to MELD, APRI, FIB-4 or ALBI score [46]. NPNT is an ECM protein that plays a role in acute hepatitis by promoting infiltration of CD4+ T cells or natural killer T cells through interaction with integrins [41]. In liver fibrosis it increases hepatic resistance by promoting HSC contraction and TGF-beta activation through interaction with α8β1 integrin [42]. According to Wanless’ hypothesis of the congestive escalator, inflammatory oedema of the liver and damage to sinusoids contribute to the development of parenchymal extinction, while microthrombosis of hepatic veins and portal branches worsens congestion and ischemia of the liver, thus contributing to the development of fibrosis. [55]. This underlines the important role of the endothelial injury and the activation of the coagulation cascade as the pathogenetic mediators of the liver injury and PH development. Syntaxin-binding protein 5 (STXBP5) is involved in endothelial exocytosis and inhibits the endothelial release of von Willebrand factor (vWF), which is otherwise elevated in PH [7,51]. Higher intensity of vWF and lower STXBP5, as demonstrated in the CSPH group in our study (S1 Table), supports the role of endothelial dysfunction as an important factor in PH development. Among the proteins with higher abundance in non-CSPH group were Annexin A2 (ANXA2) and Junction plakoglobulin (JUP). Impairment of ANXA2 can lead to thrombosis, and together with JUP has important role in maintaining endothelial cell junctions. Thus, abundance of these proteins might be considered protective against worsening PH [50,52]. PAI-1 that was identified in the serum of CSPH group of patients in our study goes in hand with the role of reactive endothelium, culminating in hemodynamic and haemostatic changes in individuals with elevated portal pressure (Fig 6) [26,34]. PAI-1 promotes thrombotic risk and can aggravate liver disease and PH by local ischemia and microthrombus formation [26]. NETs might also contribute to intrahepatic circulatory disturbance by promoting platelet aggregation, resulting in microthrombosis, congestion and subsequent parenchymal extinction [55,56]. Whereas further studies are warranted, the proposed NET-dependent effects offer an additional pathogenic pathway to the disruption of hepatic blood flow, contributing to the onset of PH as well as liver fibrosis [54,56].

The ECM accumulation is a pivotal process in the context of cirrhosis and might be considered as the reparative and adaptive response to the tissue injury that results in HSC activation and fibrogenesis [57]. It also results from the activation of the integrin-mediated pathways, as the reaction to the local haemodynamic disturbances in the liver that lead to stretching of sinusoidal endothelium [58]. We have demonstrated for the first time the abundant presence (3.5-fold increased intensity) of a disintegrin and metalloproteinase with thrombospondin motifs-like protein 4 (ADAMTSL4) in patients with CSPH. ADAMTSL4 is a secreted glycoprotein that contributes to ECM homeostasis, and was previously demonstrated to induce fibrillin-1 deposition in cirrhosis [44,45]. In the non-CSPH group we exclusively identified fibronectin, reported to mediate cell adhesion, growth, migration and ECM assembly, as well as exerting opsonin activity against bacteria. Lower levels of fibronectin have earlier been reported in patients with PH, as a consequence of increased consumption by enlarged spleen [35].

The next cluster of biological processes refers to the activation of neo-angiogenesis and lymphangiogenesis, as the adaptive and compensatory mechanisms to the liver injury and the consequent hemodynamic disturbances. The lymphatic system assumes pivotal roles in maintaining fluid homeostasis, orchestrating inflammation and immune responses [59]. Both liver fibrosis and PH are linked to lymphangiogenesis, but the molecular mechanisms governing lymphatic dysfunction in these disorders remain largely unexplored. Lymph production is elevated in cirrhosis, as a result of increased capillary permeability. The volume of hepatic lymph flow correlates with increasing portal pressure, whereas increased lymph flow stimulates hepatic lymphangiogenesis, lymph stasis and leakage [12]. Formation of new lymphatic vessels is induced by growth factors, such as VEGF-C/D, that are overexpressed along the worsening stages of liver fibrosis [60]. Our results are in line with this, as we have for the first time identified VEGF-C, but also LYVE-1, as the proteins involved in lymphangiogenesis, exclusively in the CSPH group. LYVE-1 is a major high affinity receptor for hyaluronic acid (HA) expressed by the normal wall of lymphatic vessels, LSEC and Kupffer cells, thus connecting inflammation and ECM deposition with lymphangiogenesis [27,33]. This is further supported by the interactome analysis demonstrating the interconnection between inflammation, liver fibrosis and lymphangiogenesis and their representative proteins as identified in serum of patients with CSPH (Fig 4). Additionally, TN-C, an ECM glycoprotein reported to affect lymphangiogenesis by interacting with integrins, and upregulated in lymphatic endothelial cells during lymphangiogenesis, was also abundant in serum of CSPH group in our study [38].

Cirrhosis is a consequence of chronic liver inflammation and injury, triggering a series of adaptive responses aimed at damage managing and promoting tissue repair. By interactome analysis we have revealed interesting interconnections between the most abundant proteins in CSPH centered around CD44. Its expression is triggered by HA which is elevated in liver injury [61]. CD44 is expressed by a variety of immune cells, fibroblasts, and HSCs and has a role as a proinflammatory signal that mediates the release of cytokines and promotes immune cell migration. In patients with non-alcoholic fatty liver disease (NAFLD) higher levels of circulating and liver tissue CD44 were associated with more severe liver injury [32]. Also, in a model of congestive hepatopathy CD44 has been proposed to be a marker of HSC activation [62]. While CD44 appears to have adaptive functions, its role in cirrhosis is complex and may vary depending on the specific context, necessitating additional investigation.

The graphical summary of the pathophysiology of PH development, including the key proteins involved in this process based on the results from this study and previous knowledge is depicted in Fig 6. Accordingly, we propose that **1.** Bacterial products from gut, immune complexes, and platelets trigger the formation of NETs. BPI produced by neutrophiles and released during NET formation, exerts strong antibacterial activity and neutralizes lipopolysaccharide. Although exerting antibacterial function, MPO contributes to tissue damage by reactive oxygen species (ROS) released from neutrophils. Interaction of activated leukocytes with platelets and coagulation factors may contribute to microvascular thrombosis and aggravation of PH; **2.** Damage to hepatocytes and LSEC leads to increased HA production, and in turn to CD44 mediated neutrophil sequestration, which perpetuates inflammation, HSC activation and VEGF induction, resulting in the increased portal pressure and hepatic resistance. Cell migration, ECM formation as well as thrombus formation is further supported by PAI-1, expressed by activated endothelium; **3.** ATX and NPNT influence HSC proliferation, activation, contractility and fibrogenesis. ATX also supports platelet activation and angiogenesis by activating the VEGF signalling pathway; **4.** VEGF-C released by macrophages binds to vascular endothelial growth factor receptor 3 (VEGFR-3) on the endothelial cells, triggering angiogenesis and lymphangiogenesis. LYVE-1, as a high affinity receptor for HA supports new lymph vessel growth together with CD44 and TN-C by its interaction with integrin receptors.

The main limitations of our cross-sectional study are a relatively small sample size, and pooling of the serum samples. Small sample size and single-centre nature warrants for further validation of the provided data with a larger number of patients, recruited ideally through a multicentric trial. Pooling of serum samples was chosen to put the focus on the biological processes and proteins that were most differently expressed between the two groups, including the detection of proteins which circulate at low concentrations. Whereas pooling blurs the specificity of individual samples, it provides data about the”biological average” of the group [19]. On the other hand, it is possible that a single patient with a distinct overabundant proteome pattern could skew the result of the whole group, but it is very unlikely that this happened with a large number of proteins in the pool. Thus, further validation of our data by analysing individual samples would provide firmer evidence. Another limitation arises from the fact that we purified patients’ sera using a heparin-enriched column. This technique concentrates proteins with a high heparin binding affinity. This was done deliberately in order to search for growth factors and cytokines with known heparin-binding sites, but on the other side we “ignored” part of the serum proteome with lesser heparin-binding affinity [63]. Finally, selection of the proteins highlighted in the results was based on manual curation of the literature and comparison to the existing evidence, and hence the interpretation is prone to publication bias and human error. Despite these drawbacks, our results fit well into the pool of the existing knowledge regarding the pathogenesis and worsening of PH in CLD, whilst adding the new pieces to the puzzle. Moreover, in contrast to previous reports that were limited to analysing single or a few proteins, we provide for the first-time evidence of the proteomic signature of two distinctive stages of PH in the narrowed and well characterized group of patients with cACLD.

This pilot study reveals a distinctive proteomic signature of CSPH in patients with cACLD, providing additional insights into the pathogenesis of PH development and progression in this clinical background. Based on their presence and known biological role in liver disease we propose that CD44, VEGF-C, LYVE-1, PAI-1, BPI, MPO, TN-C, NPNT, ATX and ADAMTSL4 warrant further evaluation as potential candidate biomarkers of CSPH in cACLD. Our data also indicate NET formation and inflammatory response to gut bacterial compounds, in addition to ECM, as the key steps in the development of PH, accompanied by an increased sinusoidal contractility and marked lymphangiogenesis. The protein mediators of these processes might be considered potential candidate targets for the development of a new generation of therapies.

## Supporting information

S1 Fig Functional enrichment depicting all identified biological processes associated to the proteins detected in the sera of clinically significant portal hypertension (CSPH) and non-CSPH patients, listed from smallest to largest ratio (percentage) of proteins related to the respective process. X-axis: Percentage of detected proteins associated to a specific biological process, Y-axis: Biological process identified by functional enrichment. Abbreviations: CSPH = clinically significant portal hypertension.
(TIF)

S1 Table Database with anonymous individual characteristics of the included participants. Abbreviations: BMI = Body mass index, HVPG = hepatic venous pressure gradient, LSM = Liver stiffness measurement, INR = International normalized ratio, MELD = Model for end stage liver disease, APRI = AST to platelet ratio index, FIB-4 = Fibrosis 4 index.
(XLSX)

S2 Table List of proteins identified in the CSPH and non-CSPH group and their detected intensities. Abbreviations: CSPH = clinically significant portal hypertension.
(XLSX)

S3 Table Proteins identified solely in the CSPH group, solely in non-CSPH group, and proteins with at least a 2-fold change among those present in both groups. Abbreviations: CSPH = clinically significant portal hypertension.
(XLSX)

## Abbreviations

ADAMTSL4: A disintegrin and metalloproteinase with thrombospondin motifs-like protein 4
AHSC: activated hepatic stellate cell
ALD: alcohol-associated liver disease
ANXA2: Annexin A2
APOC4: Apolipoprotein C-IV
APRI: AST to platelet ratio index
ATX: Autotaxin
BMI: body mass index
BMX: Bone marrow kinase on chromosome X
BPI: Bactericidal permeability increasing protein
cACLD: Compensated advanced chronic liver disease
CAT: Catalase
CD44: Cluster of differentiation 44
CHL1: Close homolog of L1
CLD: Chronic liver disease
COL-(1A1,1A2, 5A2, 6A3): Collagen subtypes
CSPH: Clinically significant portal hypertension
DNA: Deoxyribonucleic acid
ECM: Extracellular matrix
F13A1: Coagulation factor XIII A chain
FAM20C: Extracellular serine/threonine protein kinase
FCGR3A: Low affinity immunoglobulin gamma Fc region receptor III-A
FIB-4: Fibrosis-4 index
FLNA: Filamin-A
FN1: Fibronectin
FST: Follistatin
HA: Hyaluronic acid
HIST1 (H1B, H1E, H2BN, H4A): Histones
HSC: Hepatic stellate cells
HVPG: Hepatic venous pressure gradient
IGFBP7: Insulin-like growth factor-binding protein 7
IGF-I: Insulin-like growth factor
IGHV4-4: Immunoglobulin heavy variable 4–4
INR: international normalized ratio
ITG: Integrin
JUP: Junction plakoglobi
KC: Kupffer cells
KEGG: Kyoto Encyclopedia of Genes and Genomes
LCAT: Phosphatidylcholine-sterol acyltransferase
LC-MS: Liquid Chromatography-Mass Spectrometry
LEC: lymph vessel endothelial cell
LFQ: Label-free quantification
LPA: Lysophosphatidic acid
LPS: Lipopolysaccharide
LSEC: Liver sinusoidal endothelial cell
LSM: Liver stiffnes measurment
LYVE1: Lymphatic vessel endothelial hyaluronic acid receptor 1
MASLD: metabolic dysfunction associated steatotic liver disease
MELD: model for end stage liver disease
MetALD: metabolic dysfunction associated steatotic liver disease and increased alcohol intake
MMP2: 72 kDa type IV collagenase
MP: Macrophage
MPO: Myeloperoxidase
NAFLD: Non-alcoholic fatty liver disease
NASH: Non-alcoholic steatohepatitis
NET: Neutrophil extracellular traps
NKT: natural killer T cells
NPNT: Nephronectin
NSBB: Non-selective beta blockers
PAI-1: Plasminogen activator inhibitor 1
PH: Portal hypertension
PPIB: Peptidyl-prolyl cis-trans isomerase B
PRDX2: Peroxiredoxin-2
PTPRS: Receptor-type tyrosine-protein phosphatase S
QHSC: quiescent hepatic stellate cell
rLPA: Lysophosphatidic acid receptor
ROS: Reactive oxygen species
RWDD1: RWD domain-containing protein 1
S100: (A8, A9, A12) Protein S100
SAA2-SAA4: Serum amyloid A-4 protein
SELL: L-selectin
SERPINE1: Plasminogen activator inhibitor 1
SSC5D: Soluble scavenger receptor cysteine-rich domain-containing protein
STXBP5: Syntaxin-binding protein 5
TE: Transient elastography
TGF-β: Transforming growth factor beta
TN-C: Tenascin C
ULN: Upper limit of normal
VCAM-1: Vascular cell adhesion molecule 1
VEGF: Vascular endothelial growth factor
VEGFR3: Vascular endothelial growth factor receptor 3
vWF: von Willebrand factor
