# Supplementary figures and images for: Serum proteomic profiling of patients with compensated advanced chronic liver disease with and without clinically significant portal hypertension
Source: PLoS One. 2024 Apr 11;19(4):e0301416. doi: 10.1371/journal.pone.0301416 (PMC11008873; doi:10.1371/journal.pone.0301416)

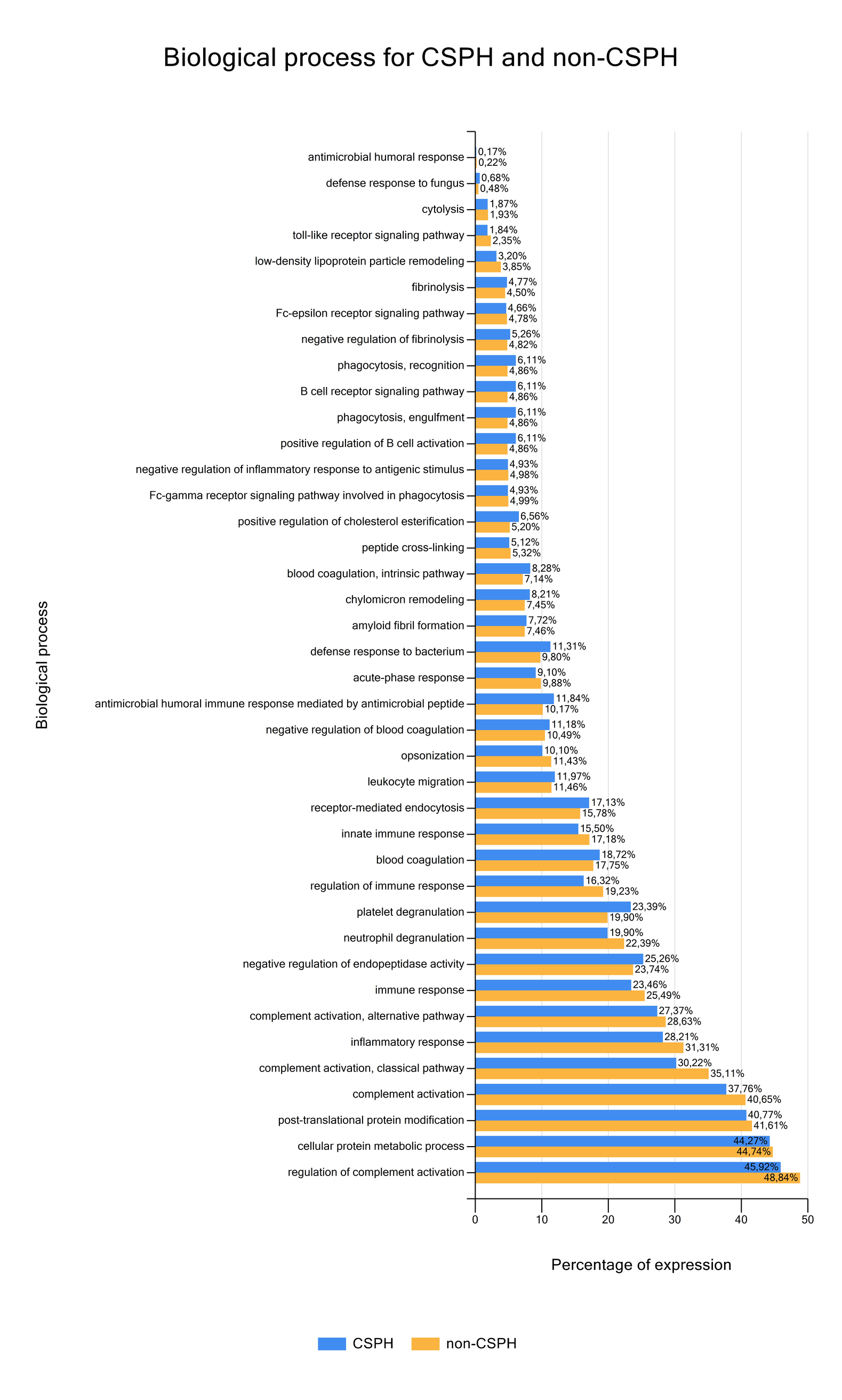

Supplement: S1 Fig — X-axis: Percentage of detected proteins associated to a specific biological process, Y-axis: Biological process identified by functional enrichment. Abbreviations: CSPH = clinically significant portal hypertension. (TIF) [file pone.0301416.s001.tif]
